# A new lizard malaria parasite *Plasmodium intabazwe* n. sp. (Apicomplexa: Haemospororida: Plasmodiidae) in the Afromontane *Pseudocordylus melanotus* (Sauria: Cordylidae) with a review of African saurian malaria parasites

**DOI:** 10.1186/s13071-016-1702-3

**Published:** 2016-08-08

**Authors:** Johann van As, Courtney A. Cook, Edward C. Netherlands, Nico J. Smit

**Affiliations:** 1 Department of Zoology & Entomology, University of the Free State, QwaQwa campus, Free State, South Africa; 2 Unit for Environmental Sciences and Management, North-West University, Potchefstroom, South Africa; 3 Laboratory of Aquatic Ecology, Evolution and Conservation, University of Leuven, 3000 Leuven, Belgium

**Keywords:** Plasmodiid taxonomy, Molecular characterisation, Morphological description, *Plasmodium zonuriae*, Haematozoa, Haemosporids, Malaria

## Abstract

**Background:**

Saurian malaria parasites are diverse apicomplexan blood parasites including the family Plasmodiidae Mesnil, 1903, and have been studied since the early 1900s. Currently, at least 27 species of *Plasmodium* are recorded in African lizards, and to date only two species, *Plasmodium zonuriae* (Pienaar, 1962) and *Plasmodium cordyli* Telford, 1987, have been reported from the African endemic family Cordylidae. This paper presents a description of a new malaria parasite in a cordylid lizard and provides a phylogenetic hypothesis for saurian *Plasmodium* species from South Africa. Furthermore, it provides a tabular review of the *Plasmodium* species that to date have been formally described infecting species of African lizards.

**Methods:**

Blood samples were collected from 77 specimens of *Pseudocordylus melanotus* (A. Smith, 1838) from Platberg reserve in the Eastern Free State, and two specimens of *Cordylus vittifer* (Reichenow, 1887) from the Roodewalshoek conservancy in Mpumalanga (South Africa). Blood smears were Giemsa-stained, screened for haematozoa, specifically saurian malaria parasites, parasite stages were photographed and measured. A small volume was also preserved for TEM studies. *Plasmodium* and *Haemoproteus* primer sets, with a nested-polymerase chain reaction (PCR) protocol, were employed to target a fragment of the cytochrome-*b* (cyt-*b*) gene region. Resulting sequences of the saurian *Plasmodium* species’ isolates were compared with each other and to other known *Plasmodium* spp. sequences in the GenBank database.

**Results:**

The presence of *P. zonuriae* in both specimens of the type lizard host *C. vittifer* was confirmed using morphological characteristics, which subsequently allowed for the species’ molecular characterisation. Of the 77 *P. melanotus*, 44 were parasitised by a *Plasmodium* species, which when compared morphologically to other African saurian *Plasmodium* spp. and molecularly to *P. zonuriae*, supported its description as a new species *Plasmodium intabazwe* n. sp.

**Conclusions:**

This is the first morphological and molecular account of *Plasmodium* species within the African endemic family Cordylidae from South Africa. The study highlights the need for molecular analysis of other cordylid *Plasmodium* species within Africa. Future studies should also include elucidating of the life-cycles of these species, thus promoting the use of both morphological and molecular characteristics in species descriptions of saurian malaria parasites.

## Background

Saurian malaria parasites are diverse apicomplexan blood parasites including the family Plasmodiidae Mesnil, 1903, and have been reported and described from a range of vertebrates since the early 1900s, see [1]. Reptilian malaria parasites have in the past been represented by four genera: *Plasmodium* Marchiafava & Celli, 1885; *Haemoproteus* Kruse, 1890; *Saurocytozoon* Lainson & Shaw, 1969; and *Haemocystidium* Castellani & Willey, 1904. However, species of *Haemoproteus* infecting reptiles (lizard, snake and chelonian) have recently been proposed to belong, on both a morphological and molecular basis, to the resurrected genus *Haemocystidium* during the revision of the haemoproteid genera by Pineda-Catalan et al. [2]. These genera, as well as the subgenera *Sauramoeba* Garnham, 1966; *Carinamoeba* Garnham, 1966; *Lacertamoeba* Telford, 1988; *Paraplasmodium* Telford, 1988; *Asiamoeba* Telford, 1988; *Garnia* Lainson, Landau & Shaw, 1971; and *Ophidiella* Garnham, 1966, were in the past solely differentiated by the morphology of the erythrocytic stages and development in the tissues of the vertebrate host, as well as by the development in the haematophagous vector [1]. The first saurian *Plasmodium* species, *Plasmodium agamae* Wenyon, 1909 and *Plasmodium mabuiae* Wenyon, 1909, were described by Weynon (1909) from an agamid lizard (*Agama agama*) and from a scincid lizard (*Trachylepis quinquetaeniata*) in Africa, respectively. On a global scale, *Plasmodium* species descriptions remained rare until the 1960s when research on these organisms greatly increased [3, 4]. Today, at least 27 species of *Plasmodium* are recorded from African lizards, but only two species have been described from African cordylid lizards (Table 1).

**Table 1:** African saurian malaria species across the families Agamidae, Chamaeleonidae, Cordylidae, Gekkonidae, Lacertidae, Opluridae and Scincidae

Lizard host species by family	Plasmodiid species	Original host localities	Intracellular meront dimensions (L × W in μm); [LW in μm^2^]; Number of merozoites	Intracellular gametocyte dimensions (L × W in μm); [LW in μm^2^]	Gametocyte morphology	Effects of gametocytes on host cell	Reference
Agamidae							
*Agama agama* [syn. *Agama colonorum* (Linnaeus, 1758)] Other hosts: *Agama cyanogaster* (Southgate, 1970); *Agama mossambica* (Peters, 1854)	*Plasmodium* (*Sauramoeba*) *giganteum* Theiler, 1930	Gbanga, Liberia	(9–18 × 4–11) [52–165] 28−74	(9–22 × 4–10) [45–145] −	Round to elongate or bulky		[3, 27–29]
*Agama agama* Other hosts: *Acanthocercus atricollis *(Smith, 1849); *Agama hispida aculeata* (Kaup, 1827)	*Plasmodium* (*Lacertamoeba*) *agamae* (Wenyon, 1909)	Bahr-El-Ghazal Province, Sudan	(4–11 × 3–6) [12–55] 4−15	(6–19 × 3–8) [33–105] −		Hypotrophy Distortion of host cells Displacement and occasional distortion of nuclei	[3, 5, 27–32]
*Agama mossambica*	*Plasmodium* (*Lacertamoeba*) *mossambica* Telford, 2009	Morogoro Region, Tanzania	(5–15 × 3–7) [20–75] 6−34	(6–17 × 3–8) [36–84] −	Elongate	Distortion of host cells Displacement and occasional distortion of nuclei Displacement of nuclei	[6]
*Agama mossambica*	*Plasmodium* (*Sauramoeba*) *giganteum* Theiler, 1930	Gbanga, Liberia	(9–18 × 4–11) [52–165] 28−74	(9–22 × 4–10) [45–145] −	Dimorphic	Hypertrophy Distortion and occasional enlargement of host cell	[27]
Chamaeleonidae							
*Chamaeleo brevicornis* Günther, 1879 Other host: *Calumma parsoni crucifer *(Cuvier, 1824)	*Plasmodium* (*Sauramoeba*) *robinsoni* (Brygoo, 1962) Telford & Landau, 1987	Moramanga Subprefecture, Madagascar	(11–23 × 7–11) [90–184] 40−74	(9–20 × 5–13) [72–221] −	Oval to elongate or bulky	Hypertrophy Displacement and distortion of nuclei	[33]
*Chamaeleo brevicornis*	*Plasmodium* (*Lacertamoeba*) *brygooi* Telford & Landau, 1987	Périnet, Madagascar	(6–9 × 5–8) [36–64] 10−16	(9–15 × 5–10) [66–126] −	Oval or elongate	Hypertrophy Distortion of cell Displacement and distortion of nuclei	[33]
*Kinyongia fischeri* (Reichenow, 1887)	*Plasmodium* (*Sauramoeba*) *acuminatum* Pringle, 1960	Tanga Region, Tanzania	− − −	− − −		Displacement of nuclei	[34]
*Kinyongia fischeri*	*Plasmodium* (*Lacertamoeba*) *fischeri* Ball & Pringle, 1965	Tanga Region, Tanzania	(9 × 6) [50] 21−25	(8–11 × 5–8) [41–87] −	Oblong to elongate	Distortion of host cell Displacement of nuclei	[35]
*Kinyongia o*x*yrhina* (Klaver & Böhme, 1988)	*Plasmodium* (*Sauramoeba*) *michikoa* Telford, 1988	Kilombero district, Tanzania	(6–15 × 4–8) [28–78] 12−32	(6–14 × 4–8) [36–80] −	Elongate	Hypotrophy	[36]
*Kinyongia o*x*yrhina*	*Plasmodium* (*Lacertamoeba*) *gologoloense* Telford, 1988	Morogoro Region, Tanzania	(5–7 × 4–6) [20–42] 6−14	(5–11 × 4–6) [20–54] −	Ovoid or round	Displacement of nuclei	[36]
*Trioceros werneri* (Tornier, 1899)	*Plasmodium* (*Lacertamoeba*) *tanzaniae* Telford, 1988	Iringa Region, Tanzania	(6–12 × 4–7) [28–70] 8−22	(8–19 × 4–9) [48–112] −	Elongate	Distortion of host cell Displacement and occasional distortion of nuclei	[36]
*Trioceros werneri*	*Plasmodium* (*Lacertamoeba*) *arachniformis* Telford, 1988	Iringa Region, Tanzania	(4–12 × 2–7) [12–49] 4−12	(6–17 × 3–8) [30–75] −	Elongate and thin	Hypertrophy Displacement and occasional distortion of nuclei	[36]
*Triceros werneri*	*Plasmodium* (*Lacertamoeba*) *uzungwiense* Telford, 1988	Iringa Region, Tanzania	(4–8 × 3–6) [16–42] 4−12	(5–13 × 3–7) [24–63] −	Elongate	Distortion of host cell Displacement of nuclei Enlargement of proerythrocyte nucleus	[36]
Cordylidae							
*Cordylus t. tropidosternum* (Cope, 1869) Other host: *Cordylus vittifer* (Reichenow, 1887)	*Plasmodium* (*Carinamoeba*) *cordyli* Telford, 1987	Tanga Region, Tanzania	(4–7 × 3–6) [12–36] 4−11	(5–8 × 4–7) [12–28] −	Round or ovoid	Hypertrophy Distortion of host cell Distortion and displacement of nuclei	[6, 7]
*Cordylus vittifer*	*Plasmodium* (*Lacertamoeba*) *zonuriae* (Pienaar, 1962)	Elandsfontein, South Africa (this study)	(undescribed); (7–17 × 4–9); (12 × 6.5) [undescribed]; [36–120]; [32–117] 18–24; 12–28; 16−24	(8–8.4 × 4.2 − 4.6); (7–20 × 4–10); (13.5 × 7) [undescribed]; [42–114]; [38–107]	Elongate	Hypertrophy Distortion and occasional enlargement of host cell Distortion and displacement of nuclei	[5] [6] This study
*Pseudocordylus melanotus* (A. Smith, 1838)	*Plasmodium* (*Lacertamoeba*) *intabazwe* n. sp.	Platberg, Harrismith, South Africa	(3.8−6.5 × 3.7−6.7) [11–26.9] 8−14	(5.5 − 7.1 × 5.4 − 6.6) [32–46] −	Kidney	Slight displacement of nuclei	This study
Gekkonidae							
*Cnemaspis barbouri* Perret, 1986	*Plasmodium* (*Lacertamoeba*) *cnemaspi* Telford, 1984	Morogoro Region, Tanzania	(6–13 × 3–7) [24–91] 8−24	(7–14 × 3–9) [32–108] −	Elongate (active) Ovoid or rounded (chronic)	Hypertrophy Distortion of host cell Displacement of nuclei	[25]
*Hemidactylus platycephalus* Peters, 1854	*Plasmodium* (*Lacertamoeba*) *uluguruense* Telford, 1984	Morogoro Region, Tanzania	(4–10 × 2–6) [12–54] 4−12	(5–10 × 4–7) [20–63] −	Ovoid	Hypertrophy Distortion of host cell Enlargement and displacement of nuclei	[25]
*Lygodactylus capensis grotei* Sternfeld, 1911	*Haemocystidium lygodactyli* Telford, 2005	University campus, Morogoro, Tanzania	− − −	(11–20 × 4–9.5) [62–140] −		Distortion of host cell	[37]
*Lygodactylus capensis grotei*	*Haemocystidium lygodactyli* Telford, 2005	Morogoro region, Tanzania	(5–10 × 9–16) [20–70] 6−26	(8–25 × 5–11) − −	Elongate to oval	Distortion of host cell	[37]
*Lygodactylus l. luteopicturatus *Pasteur, 1964 Other host: *Lygodactylus capensis grotei*	*Plasmodium* (*Lacertamoeba*) *loveridgei* Telford, 1984	Morogoro Region, Tanzania	(5–15 × 3–7) [20–91] 6−26	(8–23 × 3–11) [48–176] −	Elongate, rarely rounded	Hypotrophy Distortion of host cell Displacement of nuclei	[25]
*Tarentola mauritanica deserti* (Linnaeus, 1758) Other host: *Tarentola annularis* (Geoffroy De St-Hilaire, 1827)	*Haemocystidium tarentolae* (Parrot, 1927) Paperna & Landau, 1991	El Kantara, Algeria	− − −	(8–18 × 4–12) − −	Elongate	Slight hypertrophy and distortion of host cell Lateral displacement of nuclei.	[38, 39]
Lacertidae							
*Holaspis guentheri* Gray, 1863	*Plasmodium* (*Lacertamoeba*) *holaspi* Telford, 1986	Morogoro Region, Tanzania	(5–13 × 4–7) [25–66] 8−18	(6–18 × 3–8) [28–98] −	Elongate	Distortion of host cell Displacement and distortion of nuclei	[40]
Opluridae							
*Oplurus cuvieri* Gray, 1831	*Haemocystidium opluri* Paperna & Landau, 1991	Baie de Loukaio, Madagascar	− − −	(12–19 × 3–12) − −	Oblong, oval	Lateral hypertrophy Displacement of nuclei	[39]
Scincidae							
*Trachylepis maculilabris* (Gray, 1845)	*Plasmodium* (*Lacertamoeba*) *maculilabre* Schwetz, 1931	Kisangani, Congo	(10.0 × 6.9) [69] 15−20	(7–13 × 5–8) [42–91] −	Ovoid to elongate	Hypertrophy Distortion of host cell Displacement and occasional distortion of nuclei	[41]
*Trachylepis quinquetaeniata *(Lichtenstein, 1823) Other hosts: *Trachylepis maculilabris*; *Trachylepis striata* (Peters, 1844)	*Plasmodium* (*Carinamoeba*) *mabuiae* (Wenyon, 1909), Telford, 1983	Bahr-El-Ghazal Province, Sudan	(4–9 × 2–5 [10–30] 4−12	(5–11 × 3–5) [18–44] −	Elongate, rarely ovoid or round	Hypertrophy Occasional distortion of host cell Displacement and occasional distortion of nuclei	[31, 42]
*Trachylepis striata*	*Plasmodium* (*Sauramoeba*) *heischi* Garnham & Telford, 1984	Nairobi, Kenya	(8–18 × 6–11) [48–144] 20−65	(8–12 × 4–9) [60–120] −	Large, spindle-shaped	Distortion of host cell Lateral displacement of nuclei	[43]
*Trachylepis striata* Other hosts: *Trachylepis maculilabris*; *Trachylepis quinquetaeniata*; *Trachylepis varia* (Peters, 1867)	*Plasmodium* (*Lacertamoeba*) *pitmani* Hoare, 1932	Lake Victoria, Uganda	(4–11 × 3–7) [12–66] 4−25	(5–16 × 4–9) [25–91] − 2.07	Ovoid	Distortion of nuclei	[44, 45]

*Abbreviations*: *L*, length; *W*, width; *LW* = L × W; − Indicates dimensions not recorded

The first saurian malaria parasite from South Africa, *Plasmodium zonuriae* (Pienaar, 1962), was described from a cordylid lizard (*Cordylus vittifer*) collected at Elandsfontein (North-West province) [5]. Following the original description by Pienaar [5], an additional host, *Pseudocordylus microlepidotus* (Cuvier, 1829), collected in 1972, and locality, the vicinity of Cape Town (Western-Cape province), was recorded for this malaria parasite [6]. Later, in 1986, specimens of the type-host (*C. vittifer*) from roughly the same region as the type-locality as well as further north within South Africa (Pretoria region), were confirmed positive for *P. zonuriae*, thereby again increasing the distribution range of this parasite [6]. In his review on haemoparasites of the reptilia, Telford [6] reported a second species of *Plasmodium*, collected from South African *C. vittifer*, *Plasmodium cordyli* Telford, 1987; a parasite which he had originally described from *Cordylus tropidosternum* in Tanzania [6, 7]. Thus, by 2009, two species of *Plasmodium* had been described and reported parasitising cordylid lizards of South Africa, namely *P. zonuriae* from both *C. vittifer* and *P. microlepidotus* and *P. cordyli* from *C. vittifer* [6].

The majority of malaria parasite species descriptions in African lizards were based on the morphology of the intraerythrocytic parasites, and new species were often named based on new host infection records. This is equally true for other apicomplexan blood parasites such as the haemogregarines. However, recently there has been a concerted effort to describe known and new species of haemogregarines from African lizards using both morphological and molecular methods (see [8]). In this study we intended to do the same for three species of African saurian malaria parasites and as such the aim of this study was to morphologically confirm and molecularly characterise the two *Plasmodium* species, *P. zonuriae* and *P. cordyli*, reported infecting South African cordylids, review all existent saurian malaria parasites based on their morphometrics and describe, name and molecularly diagnose an unknown species of *Plasmodium* parasitising the South African Afromontane cordylid species *Pseudocordylus melanotus.*

## Methods

### Lizard collection, blood smear preparation and screening

Specimens of *Pseudocordylus melanotus* (*n* = 77) were collected by hand during the summer months (September to April), over a period of 5 years (2008*–*2013), at Platberg Reserve (28°14′36.71″S, 29°09′45.45″E) in the Eastern Free State Province, South Africa. Additionally, specimens of *Cordylus vittifer* (*n* = 2) were collected by hand in the Roodewalshoek Conservancy (25°00′51.20″S, 30°19′23.17″E) (1441 m), Mpumalanga, South Africa.

Blood was taken with an insulin syringe from the femoral arteries or veins and lizards were immediately released thereafter at the site of capture. Thin blood smears were prepared, air-dried, fixed and stained using Giemsa-stain (FLUKA, Sigma-Aldrich, Steinheim, Germany). Subsequently, smears were screened with the aid of a 100× oil immersion objective, images captured and parasites measured as described previously [9–12]. Morphometric measurements [length (L) and width (W) in micrometres] of the developmental stages in the lizard erythrocytes were taken using Deltapix (Nikon, The Netherlands) software. Following the method of Telford [6], the area or size was calculated as (length × maximum width) (LW) (in μm^2^) for all parasite stages examined, and the relative size of meront and gametocyte stages to the host cell nucleus (LW/HNLW) and to the nuclei of uninfected host cells (LW/NNLW) determined. A few drops of blood were fixed in 2.5 % glutaraldehyde for TEM, and the remaining blood, that was not used in blood smear preparation, was placed in sterile PCR microtubes (Eppendorf, Germany) with an equal volume of 70 % ethanol to be processed molecularly at a future date.

### Transmission electron microscopy (TEM)

Four to five drops of fresh blood were fixed in 2.5 % glutaraldehyde (0.2 M, pH 7.2) for 1 h at 4 °C. Blood was pelleted by centrifugation (10,000 rpm) and rinsed in 0.2 M Sorensen’s phosphate buffer and post fixed in 2 % solution of osmium tetroxide in the same buffer. Samples were then dehydrated in a graded ethanol series, dried over a 4Ǻ molecular sieve and embedded in Agar 100 medium (Agar Scientific, Ltd., Stansted, UK). Thin sections, cut with a glass knife on an Ultracut III Ultramicrotome (Leica, Wetzlar, Germany) were collected on copper, 300 hexagonal-mesh grids and stained for 20 min in 10 % uranyl acetate in Analar grade methanol, washed with Analar methanol and allowed to dry. Sections were then stained for 20 min in Reynolds’ lead citrate solution, washed with 0.02 M sodium hydroxide solution followed by distilled water, before examination with a Philips CM100 transmission electron microscope (Philips Electron Optics, Eindhoven, The Netherlands) operated at 80–100 kV. Digital images were captured with a MegaView II side mounted digital Olympus camera with accompanying iTEM software.

### DNA extraction and sequence analysis

Ethanol-preserved blood from the nine parasitised individuals of the 77 collected of *P. melanotus* and both *C. vittifer* were used for molecular work. Genomic DNA of *Plasmodium* spp. was extracted from the samples following the standard protocol for the Kapa Express Extract kit (Kapa Biosystems, Cape Town, South Africa). Amplification of *Plasmodium* spp. DNA was initially completed using specific *Plasmodium* and *Haemoproteus* primer sets, with a nested-polymerase chain reaction (PCR) protocol targeting a fragment of the cytochrome-*b* (cyt-*b*) gene as detailed in [13]. The PCR method consisted of two parts. The primer set HAEMNF (5′-CAT ATA TTA AGA GAA TTA TGG AG-3′) and HAEMNR2 (5′-AGA GGT GTA GCA TAT CTA TCT AC-3′) was used first under the following conditions: initial denaturation at 94 °C for 3 min, followed by 20 cycles, entailing a 94 °C denaturation for 30 s, annealing at 50 °C for 30 s with an end extension at 72 °C for 45 s, and following the cycles a final extension of 72 °C for 10 min. This was followed by a second PCR using the primer set HAEMF (5′-ATG GTG CTT TCG ATA TAT GCA TG-3′) and HAEMR2 (5′-GCA TTA TCT GGA TGT GAT AAT GGT-3′) [13]. The PCR conditions were as follows: initial denaturation at 94 °C for 3 min, followed by 35 cycles, entailing a 94 °C denaturation for 30 s, annealing at 50 °C for 30 s with an end extension at 72 °C for 45 s, and following the cycles a final extension of 72 °C for 10 min.

All PCR reactions were performed in a 25 μl volume microtube, using 12.5 μl Thermo Scientific DreamTaq PCR master mix (2×) (2× DreamTaq buffer, 0.4 mM of each dNTP, and 4 mM MgCl2), 1.25 μl of each primer, and at least 25 ng of genomic DNA for the first PCR, and 1 μl of the PCR product from the initial PCR for the second PCR. The remaining volume was made up of PCR-grade nuclease-free water (Thermo Scientific, Vilnius, Lithuania). The PCR reactions were undertaken in a Bio-Rad C1000 Touch™ Thermal Cycler PCR machine (Bio-Rad, Hemel Hempstead, UK). Resulting amplicons were visualised by means of a 1 % agarose gel stained with gel red (Biotium, USA) under UV light. The PCR products from each sample were sent to a commercial sequencing company (Inqaba Biotechnical Industries (Pty) Ltd. Pretoria, South Africa) for purification and sequencing in both directions. Resultant sequences were assembled using Geneious Ver. 7.1 (created by Biomatters. Available from http://www.geneious.com) and chromatogram-based contiguous sequences were generated, trimmed and manually corrected for ambiguous base calls. Sequences were identified using the Basic Local Alignment Search Tool (BLAST) [14], and deposited in the NCBI GenBank database under the accession numbers KX121601−KX121609.

Comparative sequences of *Plasmodium* species were downloaded from GenBank and aligned to the sequences generated within this study. Two species of *Leucocytozoon*, *Leucocytozoon gentili* and *Leucocytozoon majoris* (GenBank: DQ451435, DQ451439) were used as the outgroup, as species of *Leucocytozoon* were shown to be a sufficient outgroup of the focal taxa by [2]. All phylogenetic analyses were further undertaken in the bioinformatics software program Geneious Ver. 7.1. Sequences were aligned using the MUSCLE alignment tool [15]. To infer phylogenetic relationships a Bayesian inference (BI) method was used. A comprehensive model test was preformed to determine the most suitable nucleotide substitution model, according to the Akaike information criterion using jModelTest 2.1.7 [16, 17]. The best-fit model selected was the General Time Reversible with estimates of invariable sites and a discrete Gamma distribution (GTR + I + Γ). The dataset comprised 31 cytochrome *b* (cyt-*b*) mitochondrial sequences, with an alignment length of 497 nt. The BI analysis was implemented from within Geneious 7.1 using MrBayes 3.2.2 [18]. The Markov Chain Monte Carlo (MCMC) algorithm was run for 10 million generations. The Markov chain was sampled every 100 cycles, and the MCMC variant contained 4 chains with a temperature of 0.2. The log-likelihood values of the sample point were plotted against the generation time and the first 25 % of the trees were discarded as ‘burn-in’ with no ‘burn-in’ samples being retained. Results were visualised in Trace (implemented from within Geneious) to assess convergence and the ‘burn-in’ period.

## Results

### General observations

Parasites of what appeared to be a *Plasmodium* species were discovered in the peripheral blood of 44/77 (prevalence 57 %) specimens of *P. melanotus* captured on various rocky outcrops on the summit of Platberg Reserve in the Eastern Free State. The peripheral blood of some lizards occasionally revealed mixed infections, comprising most likely *Hepatozoon* species, filarial nematode species and so-called *Sauroplasma* infections (unpublished data)*.* Co-infections between the *Plasmodium* species and *Hepatozoon* species (of which there appeared to be possibly two species) were found in 27/77 (35 %) of the total lizards sampled. One of these *Hepatozoon* species was recently described as *Hepatozoon affluomaloti* Van As, Davies & Smit 2015, occurring in 14/77 (18 %) of the lizards [19].

**Fig. 1:**
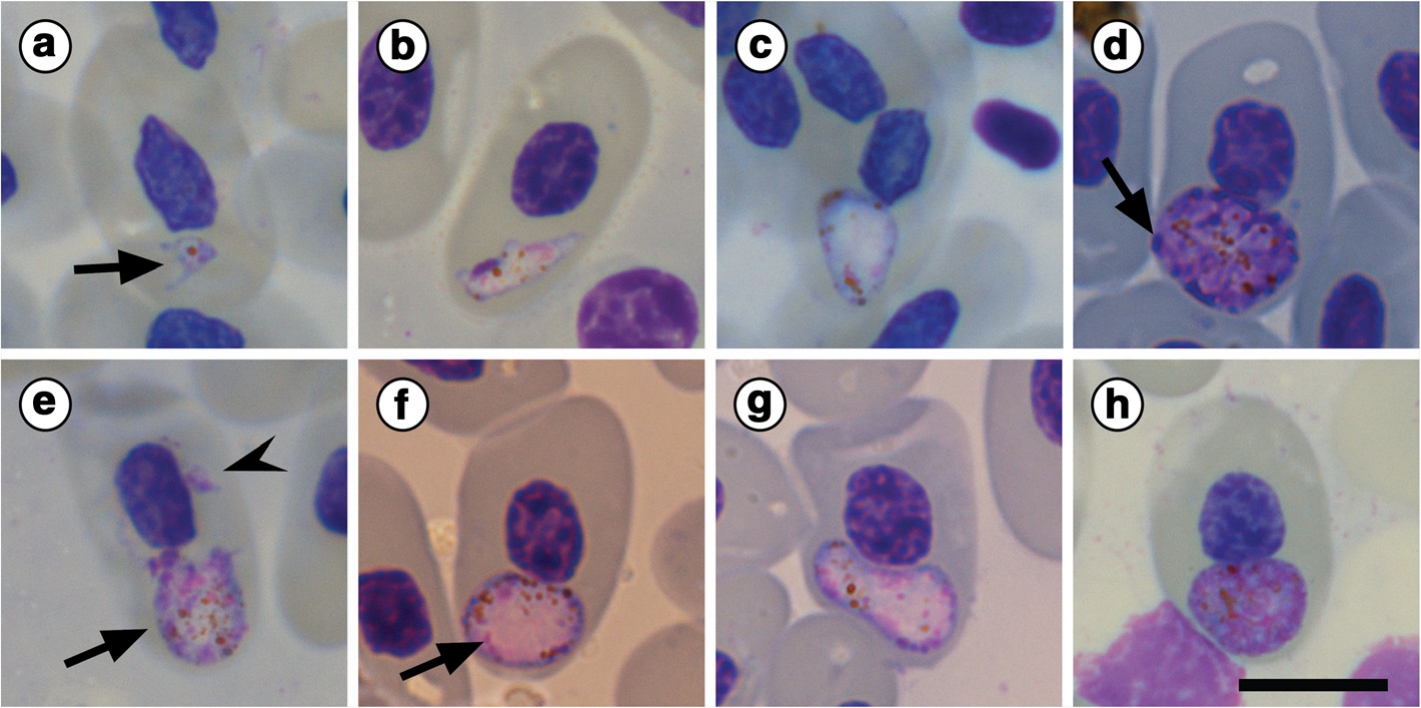
*Plasmodium zonuriae* Pienaar 1962 Telford 1987 in the lizard *Cordylus vittifer* (Reichenow, 1887) (Sauria: Cordylidae). **a** Trophozoite with pseudopodia (*arrow*). **b** Maturing elongated trophozoite. **c** Early meront. **d** Meront producing 18 merozoites (*arrow*). **e** Mature meront (*arrow*) releasing merozoites (*arrowhead*). **f** Mature meront differentiating into microgametocyte with fine malarial pigment (*arrow*). **g** Macrogametocyte with evenly distributed pigment at its periphery. **h** Macrogametocyte with peripherally arranged dark chromatin strands. Images (**a**-**h**) captured from the voucher slides (NMB P NMB P 414 and NMB P 415). *Scale-bar*: 10 μm

**Fig. 2:**
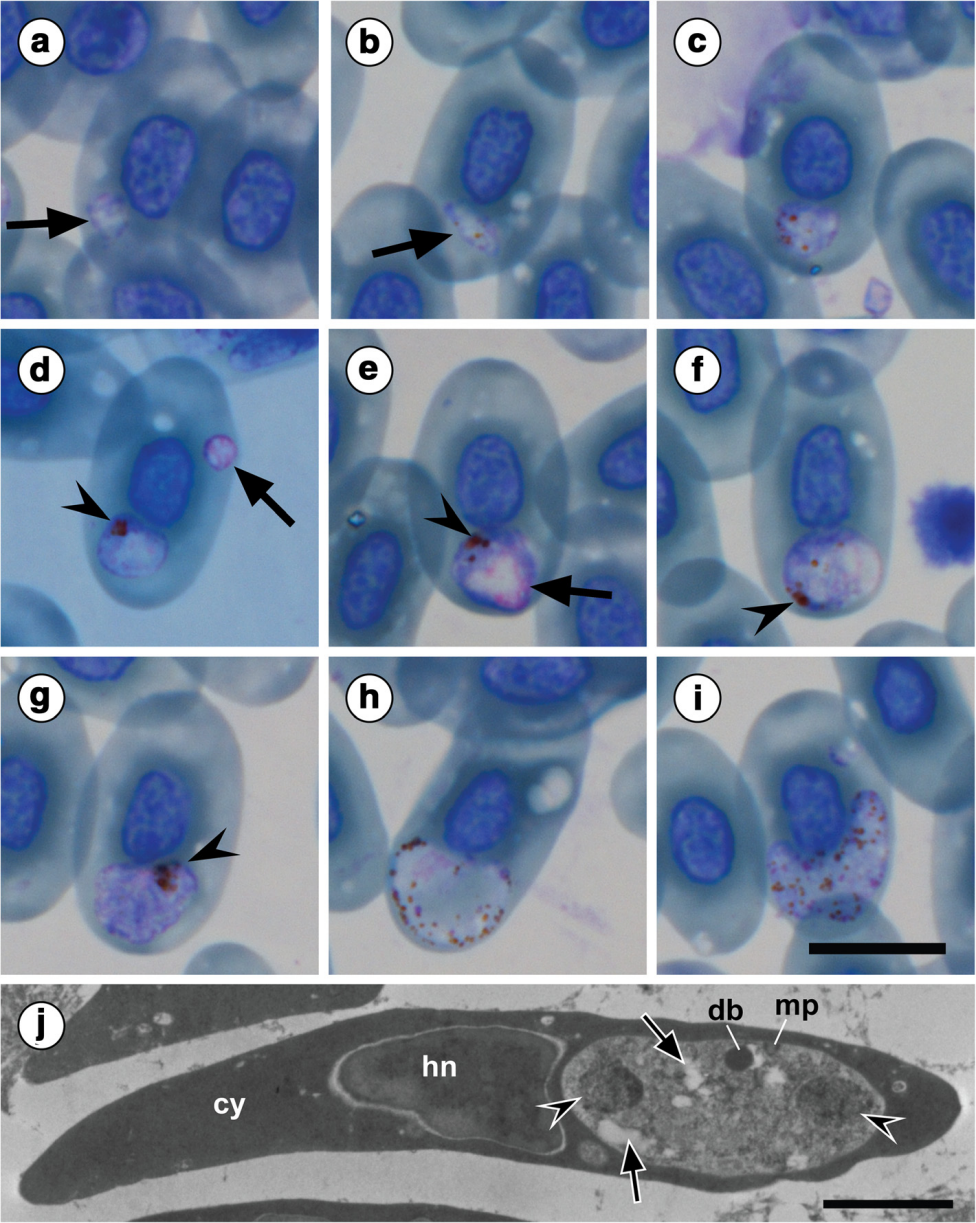
*Plasmodium intabazwe* n. sp. in the lizard *Pseudocordylus melanotus* (A. Smith, 1838) (Sauria: Cordylidae). **a** Rounded trophozoite (*arrow*). **b** Oval-shaped trophozoite (*arrow*). **c** Immature broadly rounded meront with dark golden brown pigment granules dispersed throughout. **d** Rounded meront (*arrowhead*) with rounded trophozoite (*arrow*). **e** Meront with pigment granules (*arrowhead*) producing eight merozoites (*arrow*). **f** Meront with loosely aggregating pigment granules (*arrowhead*). **g** Kidney-shaped meront with clumped pigment granules (*arrowhead*). **h** Kidney-shaped microgametocyte with irregular pigment. **i** Macrogametocyte with evenly dispersed pigment. **j** Transmission electron micrograph of an infected erythrocyte containing a meront lying in a polar position with a surface micropore (mp), various intracytoplasmic vacuoles (*arrows*), and a dense body (db). Meront membrane is in direct contact with the host erythrocyte cytoplasm (cy). Images (**a**-**i**) captured from the hapantotype and parahapantotype slides (NMB P 412 and 413, respectively). *Scale-bars*: a-i, 10 μm; j, 2 μm

*Plasmodium zonuriae* (Pienaar, 1962) infections were confirmed, based on morphological characteristics (see Table 1, Fig. 1 and Discussion), in both *C. vittifer* specimens (100 % prevalence) from the Roodewalshoek Conservancy, Mpumalanga. These infections from both lizard hosts were present in the peripheral blood erythrocytes (Figs. 1 and 2). No *Plasmodium* species, morphologically comparable to *P. cordyli*, were observed in either of the *C. vittifer* specimens or in any of the *P. melanotus* specimens.

### *Plasmodium zonuriae* (Pienaar, 1962) Telford, 1987

***Type-host*:***Cordylus vittifer* (Reichenow, 1887) (Sauria: Cordylidae).

***Vector*:** Unknown.

***Type-locality*:** Elandsfontein (North-West province) [5].

***Other localities*:** Pretoria (Gauteng) and Middelburg (Mpumalanga) [6]; Roodewalshoek (Mpumalanga) (present study).

***Voucher material*:** Voucher material, 2× blood smears from *Cordylus vittifer* NMB P 414 and *C. vittifer* NMB P 415, respectively, deposited in the Protozoan Collection of the National Museum, Bloemfontein (NMB), South Africa.

***Representative DNA sequences*:** The cyt-*b* gene sequences have been submitted in the GenBank database under the accession numbers KX121608 and KX121609.

### Redescription

***Trophozoite***. Amoeboid forms (Fig. 1a) with abundant pseudopodia, measuring 1.8–2.8 × 1.6–3.1 (2 ± 0.9 × 2.7 ± 1.4) μm (*n* = 55), containing discrete brown or black pigment granules. Oval to pyriform forms with purple-stained periphery and pinkish central vacuole (Fig. 1e*arrowhead*) measuring 1.1–1.8 × 1.0–1.7 (1.4 ± 0.3 × 1.2 ± 0.35) μm (*n* = 60); LW: 3.9–21.8 (8.4 ± 5) μm^2^ (*n* = 60). Mature trophozoites elongated (Fig. 1b), with distinct purple staining peripheral areas, 3.0–4.6 μm in diameter. Dark golden brown pigment granules dispersed throughout cytoplasm present.

***Meront***. Ovoid to pear-shaped (Fig. 1c), with slightly basophilic cytoplasm, measuring 4.6–10.2 × 4.2–7.2 (6.9 ± 1.3 × 6.2 ± 0.9) μm (*n* = 40), mostly polar in position within host cell cytoplasm. Dark golden brown pigment granules dispersed throughout meronts present (Fig. 1c), aggregating peripherally.

***Mature meront***. Polymorphic in shape (Fig. 1d-e), often slightly kidney shaped (Fig. 1d) to broadly oval (Fig. 1e), measuring 4.8–9.9 × 4.1–6.9 (5.9 ± 1.1 × 5.8 ± 0.9) μm (*n* = 33); LW: 28–49.4 (38.4 ± 7) μm^2^, with golden brown pigment granules aggregating either peripherally (Fig. 1d) or centrally within faintly eosinophilic cytoplasm (Fig. 1e). Meronts produced 12–25 (18.2 ± 3.5) (*n* = 33) pink-stained merozoites (Fig. 1d*arrow*), lying at periphery. Ruptured meronts dispersed free purple-stained merozoites within host cell cytoplasm (Fig. 1e*arrowhead*). Meront size relative to host cell nucleus (LW/HNLW): 0.7–1.4 (1.0 ± 0.2) and to normal erythrocyte nuclei (LW/NNLW): 0.9–1.6 (1.2 ± 0.2) (*n* = 50).

***Gametocyte***. Variable in shape, often oval to kidney-shaped, positioned in polar regions of host erythrocyte (Fig. 1f-g). Mature gametocytes, elongated or kidney-shaped, pale pink-stained, with fine malarial pigment (Fig. 1f*arrow*), or rounded with predominantly basophilic cytoplasm (Fig. 1f-g), producing strands of malarial pigment centrally (Fig. 1g) and peripherally (Fig. 1h).

***Microgametocyte***. Kidney-shaped, pale-staining (Fig. 1f), measuring 8.4–10.4 × 3.9–4.6 (9.9 × 4.1) μm; LW: 45.6–55.5 (52.0) μm^2^ (*n* = 18); tending to curve around host cell nucleus, sometimes slightly displacing it. Cytoplasm pale-pink, contains fine irregular brown to black pigment granules, loosely aggregating peripherally. Distinct pink staining nuclei aggregate peripherally (Fig. 1f*arrow*). Gametocyte size relative to host cell nucleus (LW/HNLW): 1.3–2.3 (1.7 ± 0.4) and to normal erythrocyte nuclei (LW/NNLW): 1.7–2.9 (2 ± 0.5) (*n* = 50).

***Macrogametocyte***. Deep basophilic staining, kidney-shaped, curving around nucleus (Fig. 1g) measuring 6.1–10.5 × 6.2–7.9 (10.1 ± 1.1 × 7.3 ± 0.9) μm (*n* = 44); LW: 52.7–91.4 (62.5 ± 16) μm^2^; with course, rounded dark-brown pigment often loosely clumped (Fig. 1g) or evenly dispersed centrally (Fig. 1h). Dark chromatin strands arranged peripherally (Fig. 1g-h). Macrogametocyte size relative to host cell nucleus (LW/HNLW): 1.6–2.0 (1.8 ± 0.2) and to normal erythrocyte nuclei (LW/NNLW): 1.4–21.8 (1.6 ± 0.2) (*n* = 50).

### Remarks

Prior to this study, only two species of *Plasmodium* had been described parasitising cordylid lizards from South Africa, *Plasmodium cordyli* and *Plasmodium zonuriae*, originally described from cordylids of Tanzania and South Africa, respectively [6]. Infections of *Plasmodium zonuriae* (Pienaar, 1962) Telford, 1987 were confirmed in the type-host lizard in a new locality Roodewalshoek in the Mpumalanga Province. Telford [6] stated that he found *P. cordyli* infecting *C. vittifer* from the then Transvaal Province (incorporating the present Mpumalanga Province), the same host and region from which *P. zonuriae* was originally described by Pienaar [5] and reported in this study. Thus it is possible that in future with the addition of more *C. vittifer* samples, we may be able to isolate *P. cordyli* for molecular analysis. Interestingly, Telford [6] described two morphological variants of *P. cordyli*, accounting for these differences as a result of parasitism of different host species.

### *Plasmodium intabazwe* n. sp.

***Type-host*:***Pseudocordylus melanotus* (A. Smith, 1838) (Sauria: Cordylidae).

***Vector*:** Unknown.

***Type-locality*:** Donkey Pass (28°16'28.99"S, 29°12'21.39"E) (2,312 m), Platberg Reserve (Free State Province).

***Other localities*:** Gibson Dam (28°16'33.12"S, 29°12'40.13"E) (2,292 m) and Platberg Pan (28°14'35.28"S, 29°09'47.02"E) (2,258 m) across the summit of Platberg, Platberg Reserve (Free State Province). 

***Type-material*:** Hapantotype, 1× blood smear from *Pseudocordylus melanotus* deposited in the Protozoan Collection of the National Museum, Bloemfontein, South Africa under accession number NMB P 412; parahapantotype, 1× blood smear from *Pseudocordylus melanotus*; deposited in the Protozoan Collection of the National Museum, Bloemfontein (NMB), South Africa, under accession number NMB P 413.

***Representative DNA sequences*:** The cyt-*b* gene sequences have been submitted in the GenBank database under the accession numbers KX121601−KX121607.

***ZooBank registration*:** To comply with the regulations set out in article 8.5 of the amended 2012 version of the International Code of Zoological Nomenclature (ICZN) [20], details of the new species have been submitted to ZooBank. The Life Science Identifier (LSID) of the article is urn:lsid:zoobank.org:pub:5BD63E9C-CA4C-4292-9AE2-E0D79169259F. The LSID for the new name *Plasmodium intabazwe* is urn:lsid:zoobank.org:act:797829AA-9E9A-4C98-B5A3-E4080DF9DBE2.

***Etymology*:** The species epithet is derived from the Zulu word for ‘flat mountain’ since the first records of this malarial parasite were found in the blood of *Pseudocordylus melanotus* collected on Platberg (Afrikaans for ‘flat mountain’) in the eastern Free State, South Africa.

### Description

***Trophozoite***. Rounded (Fig. 2a, d*arrow*) to oval (Fig. 2b*arrow*), with purple-stained periphery (Fig. 2a-b) and pinkish central vacuole, measuring 1.6–2.2 × 1.4–2.3 (1.9 ± 0.1 × 1.6 ± 0.25) μm (*n* = 30); LW: 1.4–10.4 (3.6 ± 3) μm^2^ (*n* = 50).

***Immature meront***. Broadly rounded (Fig. 2c-d), sometimes amoeboid, stained deeper pink, measuring 4.2–5.5 × 2.4–5.2 (4.9 ± 0.3 × 4.2 ± 0.7) μm (*n* = 30); LW: 6.7–20.8 (15.6 ± 4.2) μm^2^ (*n* = 50); mostly polar in position within host cells. Dark golden-brown pigment granules dispersed throughout meronts present (Fig. 2c), often aggregating at one end (Fig. 2d* arrowhead*). Immature meront size relative to host cell nucleus (LW/HNLW): 0.2–0.6 (0.5 ± 0.1) and to normal erythrocyte nuclei (LW/NNLW): 0.2–0.6 (0.5 ± 0.1) (*n* = 50).

***Mature meront***. Polymorphic in shape (Fig. 2e-f), often oval (Fig. 2e) to rounded (Fig. 2f*arrowhead*), or kidney-shaped (Fig. 2g), measuring 3.8–6.5 × 3.7–6.7 (5.2 × 5.2) μm (*n* = 18); LW: 25–44.4 (34.5) μm^2^ (*n* = 18); with irregular dark-brown or black pigment granules often loosely aggregating at periphery (Fig. 2e-g*arrowhead*). Meronts produced 8–14 pink-staining nuclei (merozoites) (Fig. 2e*arrow*), lying at periphery. TEM micrographs of erythrocytes containing *P. intabazwe* n. sp. revealed a meront lying in a polar position in an erythrocyte (Fig. 2j). The meront was sectioned so that two nuclei (merozoites) were exposed. Also observable was a surface micropore (mp in Fig. 2j), various intracytoplasmic vacuoles (Fig. 2j*arrows*), and a structure resembling a dense body (db in Fig. 2j). The meront showed no evidence of a parasitophorous vacuole, the parasite surface membrane being in direct contact with the host erythrocyte cytoplasm (cy in Fig. 2j). Mature meront size relative to host cell nucleus (LW/HNLW): 0.7–1.4 (1.1 ± 0.3) and to normal erythrocyte nuclei (LW/NNLW): 0.7–1.3 (1 ± 0.2) (*n* = 50).

***Gametocyte***. Variable in shape, often kidney-shaped, positioned in polar regions of host erythrocyte (Fig. 2h-i). Later forms, elongated or kidney-shaped, pale pink-stained, with fine malarial pigment (Fig. 2h), or deeply purple-stained, containing coarse evenly distributed dark granules of malarial pigment (Fig. 2i).

***Microgametocyte***. Kidney-shaped to loosely oval (Fig. 2h), pale-staining, tending to curve slightly around host cell nucleus, measuring 6.5–7.7 × 3.6–4.1 (6.9 × 5.4) μm (*n* = 8); LW: 47–57.5 (51.5) μm^2^ (*n* = 8); seldom causing displacement of host cell nucleus. Fine irregularly shaped, dark-brown malarial pigment tends to aggregate peripherally (Fig. 2h). Diffuse pale-pink nuclei (Fig. 2h) often clumping at extremities of cytoplasm. Microgametocyte size relative to host cell nucleus (LW/HNLW): 1.5–2 (1.7 ± 0.2) and to normal erythrocyte nuclei (LW/NNLW): 1.4–1.7 (1.5 ± 0.1) (*n* = 50).

***Macrogametocyte***. Deep staining, elongate kidney-shaped, curving around the nucleus (Fig. 2i), with evenly dispersed dark-brown pigments, measuring 5.5–7.1 × 5.4–6.6 (6.4 × 5.8) μm (*n* = 8); LW: 52.7–91.5 (62.5) μm^2^ (*n* = 8). Macrogametocyte size relative to host cell nucleus (LW/HNLW): 0.01–1.5 (1.1 ± 0.4) and to normal erythrocyte nuclei (LW/NNLW): 0.01–1.4 (1.0 ± 0.4) (*n* = 50).

### Remarks

Observation of division stages within the peripheral blood, such as meronts, indicated that this parasite belongs to the genus *Plasmodium*, as compared to the closely related *Haemocystidium*, a genus of reptile plasmodiid parasite that shows no divisional stages within the peripheral blood (see [2, 6, 9]). *Plasmodium intabazwe* n. sp. has unique morphological and morphometric features when compared to the other two *Plasmodium* spp. from cordylids, *P. cordyli* and *P. zonuriae* (see Table 1). Trophozoites and young meront stages of *P. cordyli* could not be compared with those of *P. intabazwe* n. sp. as they were not described for *P. cordyli*. Meronts of *P. intabazwe* n. sp. are similar in appearance and size to those of *P. cordyli* infecting the type-host *C. tropidosternum* in that both species demonstrate polymorphic meronts [7], measuring (mean 5.2 × 5.2 *vs* 5.5 × 4.5 μm), with merozoite numbers averaging eight and seven, respectively. However, meronts of *P. intabazwe* n. sp. do not possess the characteristic fan-shape and are not strongly nucleophilic as was reported to be evident in *P. cordyli* meronts. Notably, meronts of *P. cordyli* in the South African *C. vittifer* also lack the tendency to be nucleophilic and furthermore, the characteristic fan-shaped meronts were described as rare. Also, meronts of *P. cordyli* infecting *C. vittifer* were even larger than meronts of *P. cordyli* infecting the type-host *C. tropidosternum*, as well as being larger than the meronts of *P. intabazwe* n. sp. at (mean 7.0 × 5.6 *vs* 5.0 × 4.1 *vs* 5.2 × 5.2 μm, respectively) and contained more merozoites (mean 12 *vs* 7.5 *vs* 11, respectively). Gametocyte morphotypes of *Plasmodium cordyli* in both hosts, *C. tropidosternum* and *C. vittifer*, were reported by Telford [6] to be almost identical (dimorphic gametocytes were not reported), demonstrating rounded or ovoid gametocytes measuring on average 6.5 × 5.5 in *C. tropidosternum vs* 7.0 × 5.5 μm in *C. vittifer*, [6, 7] as compared to the kidney-shaped gametocytes of *P. intabazwe* n. sp. measuring on average 6.3 × 6 μm, which are sexually dimorphic and easily distinguishable based on pigment distribution, size and staining properties.

Microscopical examinations of the meront and gametocyte morphology led to the identification of *P. zonuriae.* Even though there was a degree of variation in the sizes of these stages in *P. zonuriae* between descriptions (see Table 1) ([5, 6], this study), there were common characteristic features. These include merozoite numbers (mean 18) and features such as the pigment granules being dispersed within both the meronts and gametocytes, and these stages curving around the host cell nucleus. Morphologically, trophozoite stages of *P. intabazwe* n. sp. and *P. zonuriae* bore a close resemblance in size, trophozoites of both species measuring on average ~1.9 × 1.6 μm. Trophozoites of *Plasmodium intabazwe* n. sp. are polymorphic as are trophozoites of *P. zonuriae*. Furthermore, early meront stages of *P. intabazwe* n. sp. also resembled those of *P. zonuriae* in size and appearance, forming shapes from oval to elongated*,* or even on occasion amoeboid shapes. However, the division stages (meronts) in the peripheral blood of the two species differ. The meronts of *P. intabazwe* n. sp. contain eosinophilic cytoplasm with fine, yet abundant pigment that aggregates peripherally or centrally as compared to the division stages of *P. zonuriae*, which contain evenly distributed pigment at the periphery. Furthermore, meronts of *P. intabazwe* n. sp. are overall smaller than the meronts of *P. zonuriae* (mean 5.2 × 5.2 *vs* 12.0 × 6.5 μm) (unfortunately these dimensions were not provided in the original description of *P. zonuriae* [5]) see [6]. In addition, meronts of *P. intabazwe* n. sp. contain eight merozoites on average (*vs* 18 in *P. zonuriae*) [5, 6]. The gametocyte stages of *P. intabazwe* n. sp. and *P. zonuriae* also differed substantially. Gametocytes of *P. intabazwe* n. sp. are sexually dimorphic and usually kidney-shaped, measuring on average 6.3 × 6.0 μm, and differ from *P. zonuriae*, which are usually elongated or rounded, measuring on average 10.9 × 5.7 μm ([5, 6], this study). Although microgametocyte stages of *P. intabazwe* n. sp. may at times resemble those of *P. zonuriae* in appearance and size, the pigment granules were in general much finer and evenly dispersed ([5, 6], this study)*.*

At a cytopathological level, erythrocytes infected with *P. intabazwe* n. sp. were neither hypertrophied nor dehaemoglobinized and rarely stained paler than non-infected cells. On the other hand, infections with *P. zonuriae* in *C. vittifer* are reported to have caused severe anaemic changes in the peripheral blood, causing hypertrophy, and displacing and distorting host cell nuclei [5, 6]. However, these effects may not always be present, as reported for the meronts of *P. zonuriae* infecting its other host *P. microlepidotus* from the Western Cape Province, which caused little distortion of either the host cell or its nucleus. Similarly, *P. intabazwe* n. sp. also showed little effect on host cells, but in some cases meronts and gametocytes tended to curve around the host cell nucleus, though they never displaced or deformed it.

### Molecular analysis

Amplicons of 500 nt from *Plasmodium zonuriae* and *Plasmodium intabazwe* n. sp., were derived from the blood of two *C. vittifer* and seven *P. melanotus*, respectively. Sequences isolated in the current study from *P. zonuriae* and *P. intabazwe* n. sp. confirmed the morphological observations by representing two distinct morphotypes, with an interspecific divergence of 3.4 % based on 497 nt sequence comparisons (percentage of bases/identical residues). For the phylogenetic analysis, only a single nucleotide sequence from both *P. zonuriae* and *P. intabazwe* n. sp. was used, as all the nucleotide sequences obtained in the current study from both *P. zonuriae* (*n* = 2) and *P. intabazwe* n. sp. (*n* = 7) were identical. These sequences were analysed together with sequences for 18 *Plasmodium* spp., four *Haemoproteus* spp., five *Haemocystidium* spp. and two *Leucocytozoon* spp. (used as the outgroup), all downloaded from the GenBank database (see Table 2).

**Table 2:** List of organisms used in the phylogenetic analyses of this study according to associated host group, with associated GenBank accession numbers, host and references

Group	Accession number	Parasite	Host	Reference
*Plasmodium* spp. of lizards	AY099061	*P. chiricahuae*	*Sceloporus jarrovi*	[46]
	KR477583	*P. fairchildi*	*Norops cupreus*	[47]
	AY099059	*P. floridense*	*Anolis oculatus*	[46]
	EU834707	*P. gemini*	*Hypsilurus modestus*	[48]
	KX121601	*P. intabazwe* n. sp.	*Pseudocordylus melanotus*	Current study
	EU834704	*P. koreafense*	*Sphenomorphus jobiensis*	[48]
	EU834710	*P. lacertiliae*	*Emoia longicauda*	[48]
	EU834705	*P. megalotrypa*	*Sphenomorphus simus*	[48]
	AY099060	*P. mexicanum*	*Sceloporus occidentalis*	[46]
	EU834703	*P. minuoviride*	*Prasinohaema prehensicauda*	[48]
	KX121608	*P. zonuriae*	*Cordylus vittifer*	Current study
*Plasmodium* spp. of birds	JN164734	*P. circumflexum*	*Sylvia atricapilla*	Unpublished
	AF069611	*P. elongatum*	*Passer domesticus*	[49]
	AY099029	*P. gallinaceum*	*Gallus gallus*	[46]
	DQ659553	*P. relictum*	*Hemignathus virens*	[50]
*Plasmodium* spp. of primates	AF069605	*P. falciparum*	*Homo sapiens*	[49]
	AF069621	*P. knowlesi*	Old World monkeys	[49]
	AF069624	*P. malariae*	*Homo sapiens*	[49]
	AF069610	*P. reichenowi*	*Pan troglodytes*	[49]
	AF069619	*P. vivax*	*Homo sapiens*	[49]
*Haemoproteus* spp.	DQ630008	*H. balmorali*	*Muscicapa striata*	[22]
	DQ630010	*H. lanii*	*Lanius collurio*	[22]
	DQ630005	*H. pallidus*	*Ficedula hypoleuca*	[22]
	JN164722	*H. parabelopolskyi*	*Sylvia atricapilla*	Unpublished
*Haemocystidium* spp.	AY099062	*H. kopki*	*Teratoscincus scincus*	[46]
	KF049514	*H. mesnili*	*Naja annulifera*	[2]
	KF049506	*H. pacayae*	*Podocnemis unifilis*	[2]
	KF049492	*H. peltocephali*	*Podocnemis expansa*	[2]
	AY099057	*H. ptyodactylii*	*Ptyodactylus hasselquistii*	[46]
*Leucocytozoon* spp. (outgroup)	DQ451435	*L. gentili*	*Passer domesticus*	[51]
	DQ451439	*L. majoris*	*Fringilla coelebs*	[51]

**Fig. 3:**
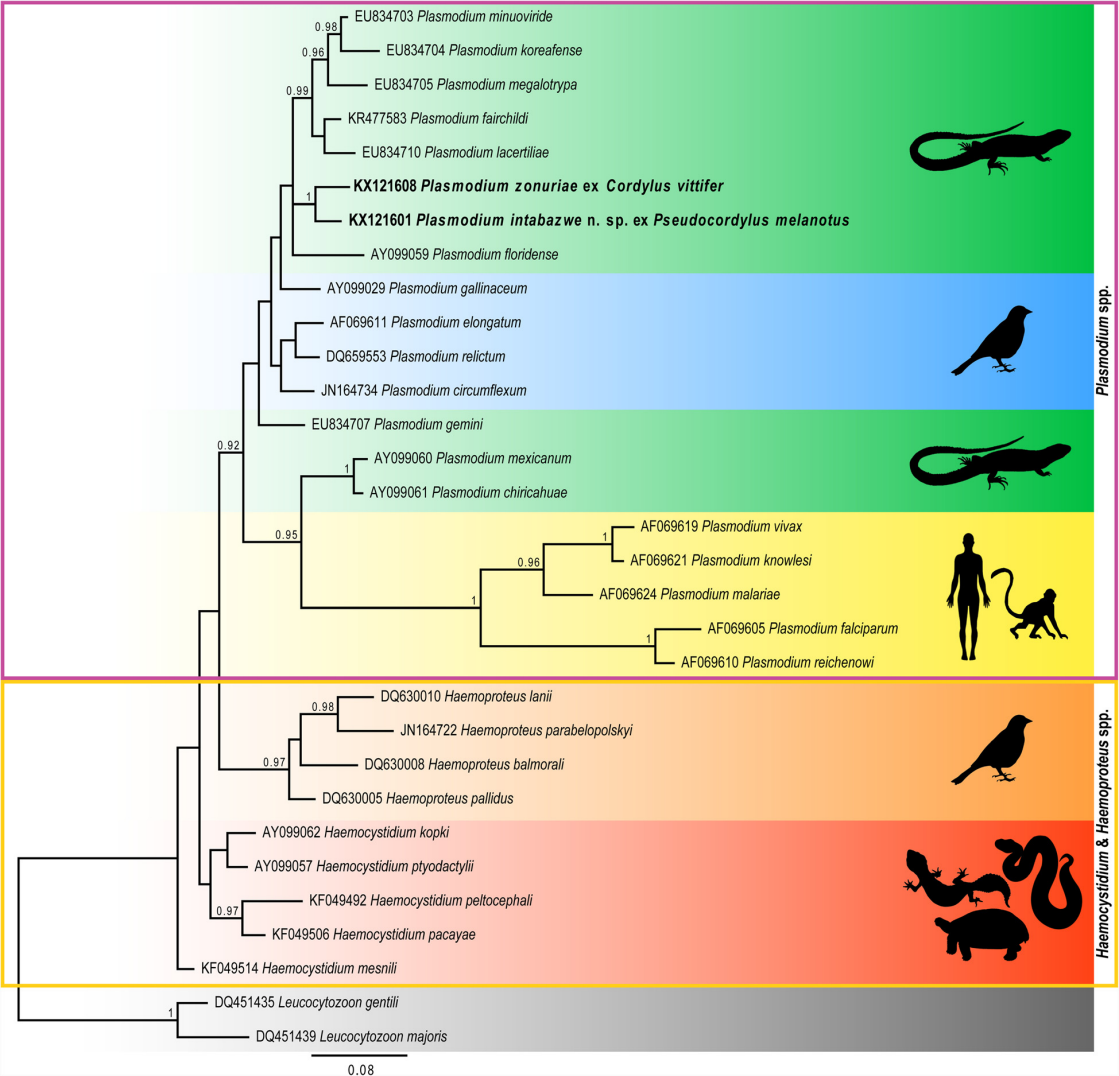
Phylogenetic analysis of *Plasmodium* spp. and closely related protozoans based on cyt-*b* gene sequences including sequences for *Plasmodium intabazwe* n. sp. and *Plasmodium zonuriae* generated in the present study (*highlighted in bold*). Bayesian inference (BI) analysis showing the phylogenetic relationships for 20 *Plasmodium* species, four *Haemoproteus* species, five *Haemocystidium* species and two *Leucocytozoon* species (used as the outgroup). All comparative sequences were downloaded form GenBank. Nodal support is provided by posterior probability values

The BI tree (Fig. 3) showed *Plasmodium* species isolated from lizard and bird hosts to be polyphyletic. *Plasmodium* species isolated from primate hosts were shown to form a well-supported monophyletic clade, forming a sister clade to *Plasmodium chiricahuae* (AY099061) and *Plasmodium mexicanum* (AY099060) isolated from North American lizard hosts. *Haemoproteus* species isolated from bird hosts formed a well-supported monophyletic clade separate from *Plasmodium* and *Haemocystidium* species. At the base of the phylogeny *Haemocystidium* species isolated from reptilian hosts were shown to be paraphyletic. The phylogenetic analysis confirmed the generic placement of the two species, *P. zonuriae* and *P. intabazwe* n. sp., within a large monophyletic clade consisting of only *Plasmodium* species. The two species from the current study were also shown to be closely related, forming a monophyletic clade with strong nodal support. Furthermore, these sequences clustered with *Plasmodium* species isolated from other lizard hosts.

## Discussion

*Plasmodium intabazwe* n. sp. can be distinguished from other African cordylid malarial parasites, both morphologically in terms of not only its morphometric characteristics, but also its staining properties, distribution of pigment and cytopathology.

Even though the morphological findings strongly suggested that *P. intabazwe* n. sp. is a different species to both *P. cordyli* and *P. zonuriae*, *P. intabazwe* and *P. zonuriae* did share similar morphological characteristics for a number of their peripheral blood life stages. This highlights the importance, when possible, of using both morphology and molecular characteristics in species descriptions. Even in cases where the morphology of two potential species is different, it is important to use molecular methods to differentiate them. As emphasized by Perkins [21], morphology of parasites can alter depending on the processing conditions of the slides, never mind the potential plasticity of the traditional characteristics used to differentiate these organisms. Unfortunately, the morphological findings could not be supported molecularly for *P. cordyli*. It would have been preferable to have molecularly compared *P. cordyli* to both the other species. However, if found in future through more extensive sampling, we would still be cautious in characterising this species as *P. cordyli* given that *C. vittifer* and South Africa are not the type-host or locality and given the morphological differences between the two variants.

The morphological findings regarding *P. zonuriae* and *P. intabazwe* n. sp. were further supported by the molecular data obtained for both species with an interspecific divergence of 3.4 %. Although [22] and [23] have suggested that the mean ‘cut-off’ for distinguishing between different *Plasmodium* and *Haemoproteus* species is an interspecies divergence of 5 %, a number of species such as *Haemoproteus pallidus* Valkiūnas & Iezhova, 1991 and *Haemoproteus minutus* Valkiūnas & Iezhova, 1992, with an interspecific divergence of approximately 1 % in sequences of the cyt-*b* gene, have been shown to be separate species when combining both morphological and molecular data (see [22, 24]).

In the phylogenetic analysis, *P. zonuriae* and *P. intabazwe* n. sp. were well nested within a larger monophyletic clade comprising only *Plasmodium* species. Although this clade contained moderate posterior probability support, the overall tree topology was similar to other studies such as [24], and particularly with a review on the closely related haemoprotid genera (see [2]). In the latter study, although the *Haemoproteus* spp. clade was shown to be the basal clade, the nodal support values separating the major *Plasmodium* species, *Haemocystidium* species and *Haemoproteus* species clades were all considerably higher. This was most likely attributable to the multigene and concatenated approach that was used for the phylogenetic analysis by [2], as compared to the current study’s cyt-*b* gene analysis, which is expected to provide a less robust analysis due to its variability.

Outside South Africa, and other than *P. cordyli* in Tanzania [7], *P. intabazwe* n. sp. is perhaps morphologically closest to *Plasmodium uluguruense* Telford, 1984 described from the gecko *Hemidactylus platycephalus* in the Uluguru Mountains, Tanzania [25]. Meronts of *P. uluguruense*, however, were more variable in size and measured on average 7 × 4 μm, while meronts of *P. intabazwe* n. sp. were more constrained in their dimensions (mean 5.2 × 5.2 μm). The gametocytes of the two species also differ, with those of *P. uluguruense* being larger and ovoid (mean 7.5 × 5.5 μm), the pigment also not dispersed within the gametocytes, but rather forming an aggregate of dark greenish-yellow granules near the cell margin of both sexes of gametocytes. The smaller, microgametocytes of *P. intabazwe* n. sp. had distinct pigment distributed around the periphery of the parasites, measuring (6.0–7.9 × 3.5–5.6 μm). Microgametocytes of *P. uluguruense* were reported to be longer and larger than the macrogametocytes, measuring on average 8 × 5 μm [6].

Interestingly, as reported for *P. microlepidotus* during Pienaar’s [5] study, in this study *P. melanotus* were also found to be parasitised by the prositigmatic mites *Zonurobia semilunaris* [26], although they occur in disjunctive localities. These mites were the only haematophagous invertebrates observed feeding on *P. melanotus*, warranting further study in future for developmental stages of *P. intabazwe* n. sp.

## Conclusions

The molecular characterisation of *P. intabazwe* n. sp. along with that of *P. zonuriae* represents the first combined morphological description and molecular characterisation of South African saurian malarial parasites, additionally confirming their placement within *Plasmodium*. The detection and description of the sporogonic stages in the natural vector should also be a key consideration in future species descriptions. Future research should include the identification of possible definitive hosts or vectors such as mites and mosquitoes as well as experimental transmission studies. Most importantly however, this work increases biodiversity knowledge of saurian malarial parasites, which by adding to the known taxa, contributes to further studies into these organisms’ taxonomy and phylogeny.
